# The diverse roles of insulin signaling in insect behavior

**DOI:** 10.3389/finsc.2024.1360320

**Published:** 2024-04-04

**Authors:** Anastasia A. Weger, Clare C. Rittschof

**Affiliations:** Department of Entomology, University of Kentucky, Lexington, KY, United States

**Keywords:** nutrition, developmental plasticity, fat body, foraging, fecundity, mating, social insect, genetic tool kit

## Abstract

In insects and other animals, nutrition-mediated behaviors are modulated by communication between the brain and peripheral systems, a process that relies heavily on the insulin/insulin-like growth factor signaling pathway (IIS). Previous studies have focused on the mechanistic and physiological functions of insulin-like peptides (ILPs) in critical developmental and adult milestones like pupation or vitellogenesis. Less work has detailed the mechanisms connecting ILPs to adult nutrient-mediated behaviors related to survival and reproductive success. Here we briefly review the range of behaviors linked to IIS in insects, from conserved regulation of feeding behavior to evolutionarily derived polyphenisms. Where possible, we incorporate information from *Drosophila melanogaster* and other model species to describe molecular and neural mechanisms that connect nutritional status to behavioral expression via IIS. We identify knowledge gaps which include the diverse functional roles of peripheral ILPs, how ILPs modulate neural function and behavior across the lifespan, and the lack of detailed mechanistic research in a broad range of taxa. Addressing these gaps would enable a better understanding of the evolution of this conserved and widely deployed tool kit pathway.

## Introduction

Nutritional state is a universal factor that alters behavioral expression in animals including insects (1, 2). Adult insects must accrue sufficient energy to support things like somatic maintenance, mate search, egg development, nest construction, oviposition, and parental care (3–7). To do this, individuals combine complex information about their own nutritional state with environmental information like resource and mate availability (8) in order to make prudent decisions about energy acquisition and use.

Insulin/insulin-like growth factor signaling (IIS) is one of the most well-recognized pathways that contributes to the organization and expression of energy-sensitive behaviors (1, 9). This pathway, particularly its satiety signaling function, is conserved across vertebrates and invertebrates (9). However, presumably because of the diverse connections between nutritional state and behavioral expression, IIS has been co-opted to regulate phenotypes like egg production, reproductive tactics, and courtship behavior across taxa (10–12). It thus offers fertile ground for studies that investigate the physiological links between nutritional state and nervous system processes, and how these relationships evolve.

In this mini review, we explore the variety of roles for IIS in regulating behavioral expression in adult insects. One of our major goals is to describe links between IIS activity and the modulation of nervous system function, highlighting knowledge gaps in these areas. To do so, we use known mechanistic examples from *Drosophila melanogaster* (13, 14), and draw parallels and distinctions with other species where possible. To emphasize the expansion and diversification of IIS over evolutionary time, we focus on behaviors ranging from most conserved (e.g., feeding behaviors) to derived (e.g., social behaviors and polyphenisms).

## Insulin/insulin-like growth factor signaling pathway fundamentals

IIS activity is dynamic throughout life. Here we focus on how IIS modulates adult behaviors, but we include some developmental processes that give rise to adult polyphenisms. IIS involves the action of insulin-like peptides (ILPs), which are produced in the brain and peripheral tissues and operate either as circulating hormones or neuromodulators (15–17). These peptides fall into three categories based on their shared homology with their vertebrate counterparts: insulin-like, insulin growth factor-like (IGF), or relaxin-like (18). Most ILPs are insulin-like (18, 19). Studies in some taxa differentiate insulin-like, IGF-like, and relaxin-like peptides, but many others refer to all types collectively as ILPs (18, 20, 21). In keeping with the convention set by *D. melanogaster*, we will generally refer to ILPs but note IGF and relaxin-like peptides where possible.

Insulin-like and IGF-like peptides activate the tyrosine kinase insulin receptor (InR) causing insulin receptor substrate (IRS) phosphorylation and downstream activation or inhibition of effectors via two major pathways, the phosphoinositide 3-kinase/protein kinase b (PI3K/Akt) pathway, which is associated primarily with cellular energy metabolism (22, 23), and the mitogen-activated protein kinase (MAPK) pathway, which is involved in cell and organismal growth, typically during development, via ecdysone signaling (4, 20). Notably, these pathways can have overlapping effects that are difficult to differentiate (18, 24–26). With PI3K/Akt, IRS binds to PI3K, activating Akt, which phosphorylates and inhibits a class O of forkhead box transcription factor (FOXO) and its downstream targets (27, 28), including developmental growth and differentiation regulators in conserved pathways such as hedgehog signaling (29–31, see 32 for an example of FOXO activity in adults). Akt can also activate the cAMP-response element binding protein (CREB, involved in memory formation) and inactivate glycogen synthase kinase 3 (GSK3), promoting glycogen synthesis and energy storage (23, 33–35). Alternatively, IRS can interact with growth factor receptor bound protein-2 (Grb2), ultimately initiating MAPK signaling (23).

While the identity of insulin-like peptides and IGFs are well-established in a variety of insect species, less is known about relaxin-like peptides outside of *D. melanogaster* (36). In *D. melanogaster*, relaxins activate G-protein coupled receptors (GPCRs), specifically leucine-rich repeat-containing GPCRs 3 and 4 (Lgr3 and Lgr4) during metamorphosis and oviposition, respectively (14, 18, 37–40). Recent studies are beginning to investigate relaxin-like peptide GPCRs in other taxa (24, 37, 41, 42).

**Figure 1:**
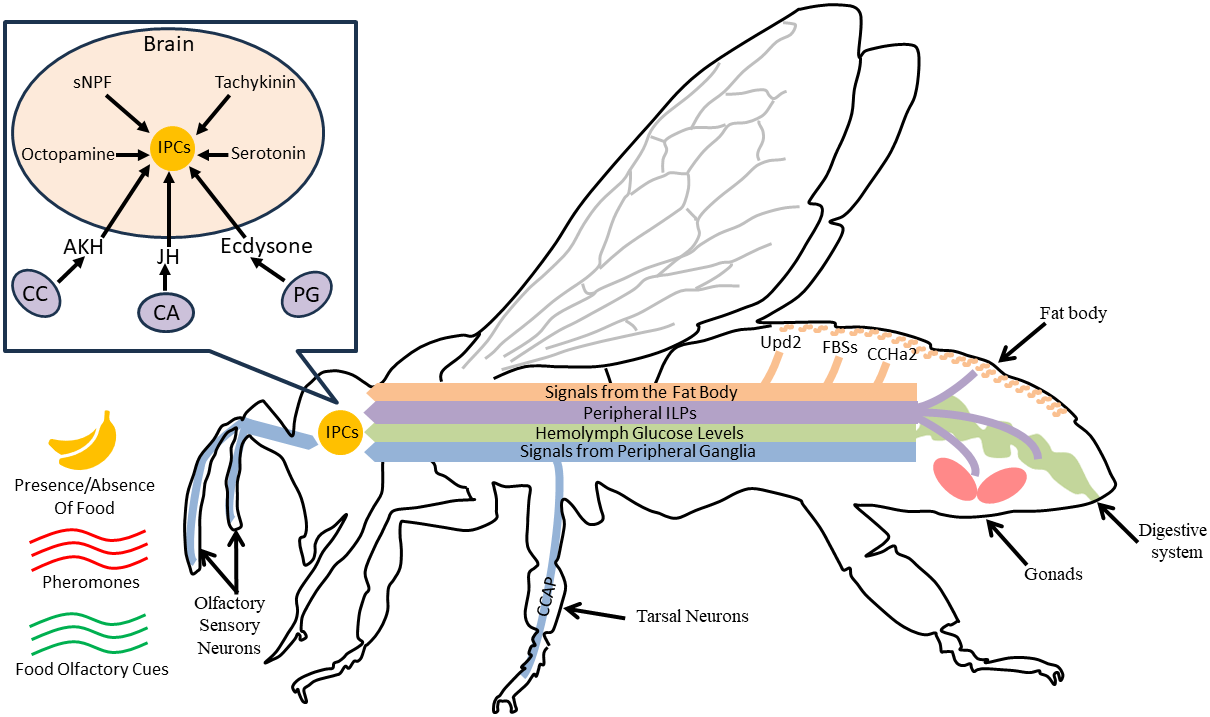
For a hypothetical adult insect, we show various IIS mechanisms that coordinate activity in the brain and periphery to give rise to behavioral variation. In the head (box insert), IPCs release locally acting ILPs to modulate nervous system processes like sensory responses and locomotor activity. Nearby glands such as the corpora cardiaca (CC), corpora allata (CA), and the prothoracic gland (PG) produce hormones that can alter ILP production and release from IPCs (18, 47). IPCs are activated by peptides (sNPF, tachykinin) or biogenic amines (octopamine, serotonin) released by other neurons in the brain in response to neural or peptide signaling from peripheral sensory systems (navy blue lines, (48, 49), or peripheral signals like hemolymph glucose levels (green line, 46); these are modulated by social and nutritional cues and nutritional status (indicated by navy blue lines, 18). The fat body also releases several types of uncharacterized fat body signals (FBSs), as well as Upd2 and CCHa2 in response to changes in available nutrients, and these ultimately stimulate IPC ILP production through unknown mechanisms (17). The fat body, and other tissues including the midgut and gonads (e.g., ovaries), also produce ILPs, shown in purple (15). These ILPs, some of which are also produced by the IPCs, can act on the brain as well as ganglia or other peripheral tissues (15). Notably, although we have depicted all relationships with a directional arrow, various signals can activate or inhibit IPCs depending on environmental context and the specific taxa.

IIS activity is often manipulated and/or measured using changes in ILP, InR, or IRS mRNA or protein levels. FOXO mRNA levels are also commonly used to infer PI3K/Akt pathway activity (43); other downstream effects of InR and the effects of relaxin-like peptides are less studied. To understand the role of IIS in coordinating nutritional state and behavior, it is necessary to know the location of ILP production and action in the periphery and brain. These are best understood in *D. melanogaster* (reviewed in 15), although characteristics are likely to be similar in other species (9, 44). In *D. melanogaster*, some ILPs are released by insulin-producing neurosecretory cells (IPCs) in the brain, where they act locally (45). IPCs respond directly and indirectly to peripheral signals including fat body produced ILPs, hemolymph glucose content, adipokinetic hormone, and other peptides and biogenic amines that can also act independently of nutritional state (17, 18, 37, 46, **Figure 1**). IPCs project to the heart, corpora cardiaca, and the midgut, stimulating ILP release from those tissues (8, 18, 50). Peripheral ILPs are also produced by ovarian follicle cells and regions of the gut. Some of these ILPs act locally, and others circulate (15, 51, 52). Notably, ILP production and inhibition are impacted by circulating hormones including juvenile hormone (JH) and ecdysteroids, and in turn, ILPs can affect the synthesis of these hormones (38, 47, 53–56). Many details regarding the coordination of ILP production and release among tissues, and the interaction of IIS with other behaviorally relevant pathways, are still under study.

## IIS regulation of feeding behavior

Perhaps the most universal function of IIS is in satiety signaling, telling an individual they do not need food (46). IIS activity has been implicated in feeding behaviors in diverse species, including fruit flies (*D. melanogaster*), locusts (*Schistocerca gregaria*), and mantids (*Tenodera sinensis* 9, 44, 57). ILPs produced in brain IPCs or peripherally, e.g., in the fat body, alter sensitivity to food cues or food acquisition behaviors through changes in sensory physiology, activity levels, nutrient preferences, and learning and memory processes (1, 9, 58–61). For example, in *D. melanogaster*, elevated circulating ILPs following food intake inhibit short neuropeptide F (sNPF) expression in the olfactory sensory neurons, reducing sensitivity to food odors and inhibiting food searching behavior (45). Similarly, starvation, and decreased production of ILPs by IPCs, induces hyperactive food search (62), while locomotion inhibits IPC ILP production, increasing sensitivity to food cues (63).

Data from other insects indicate that at least some IIS-mediated satiety mechanisms are generally conserved, although locations of ILP production, signaling relationships, and neural mechanisms giving rise to behavioral variation may differ. For example, in the desert locust *Schistocerca gregaria*, IIS via both MAPK and PI3K/Akt pathways increases sNPF expression in the optic lobe, leading to decreased feeding (57). Parasite infection at the time of a bloodmeal increases mosquito (*Anopheles stephensi*) olfactory sensitivity to hosts due to changes in midgut ILP mRNA expression (59). Female mosquitoes alternate between nectar and blood feeding as their nutritional needs change with egg production. In the mosquito *Aedes aegypti*, nutrient-specific hormone dynamics stimulate different sets of ILPs in the brain and peripheral tissues to synchronize metabolism and reproductive stage (47), as well as activate digestion of blood meals along with the target of rapamycin (TOR) pathway (64). In Western honey bees (*Apis mellifera*), IIS in the fat body modulates neural sensory systems via unknown mechanisms to cause a preference for lipid and protein-rich pollen over nectar in foragers (65). In this species, increased expression of brain InR mRNA is also correlated with spatiotemporal memory formation and anticipation of encountering known food resources (66), possibly through the MAPK pathway (67). Similarly, in *D. melanogaster*, IIS has been linked to cAMP-dependent memory formation and aversive learning in both adults and larvae (34, 68–71). In mantids, injection of mammalian insulin causes decreased movement, but rather than decreasing foraging activity, this causes a shift from active prey stalking to a more sedentary ambush strategy (44). It is largely unknown how ILPs modulate nervous system processes in these diverse species and contexts, but clearly IIS is involved in many types of behaviors and preferences related to foraging and diet choice.

## IIS regulation of courtship, mate choice, and oviposition

In insects, IIS reflects nutrient availability for reproduction, and as such it affects vitellogenesis and the number of eggs a female produces (43, 72). However, because reproductive individuals perform suites of behaviors required to successfully mate and lay eggs, IIS is also more broadly involved in courtship and mate choice (49, 73). For example, in *D. melanogaster* males, tarsal contact with pheromones from male competitors or heterospecific females leads to the release of an ILP from the IPCs, inhibiting the P1 neurons that promote courtship (49). Relaxin-like ILPs and associated downstream mechanisms in male glial cells and abdominal ganglion neurons are also required for mating, sexual receptivity, and mate attraction in *D. melanogaster* (74–76). Similarly, in females, IIS in olfactory sensory neurons responsive to male sex pheromones mediates a starvation-induced decrease in sexual receptivity (12, 48). IIS seems to incorporate individual mating history in the context of mating decisions: inhibiting ILP production in unmated females increases sexual receptivity (77) while following a mating event, decreased InR expression or ILP production reduces willingness to remate (78).

Peripheral IIS activity in females also alters attractiveness to males through cuticular hydrocarbon (CHC) profiles. In *D. melanogaster*, increased ovary IIS with decreased fat body IIS alters CHC production in fat body oenocytes (73) and increases mate attraction (52, 79). Because diet and nutrition influence IIS and CHC production, CHCs are honest signals of female quality (80). CHCs can indicate female mating status, fertility, and mating compatibility in many other insect species, suggesting this connection between IIS and mating cues may be broadly conserved in species from hymenopterans to coleopterans (81–83).

IIS mediates maternal offspring provisioning and oviposition site selection, combining the classic role of IIS in feeding behavior with its more elaborated reproductive functions. *D. melanogaster* females use gustatory cues to choose oviposition sites based on substrate sucrose concentrations (40). Interestingly, these decisions are not mediated by IPC-produced ILPs, but rather via relaxin-like ILP7 activity in neurons in the thoracic-abdominal ganglia (15), which have projections to the sub-esophageal ganglia and the female reproductive tract (40). The other *D. melanogaster* relaxin peptide, ILP8, is expressed in follicle cells and binds to receptors on abdominal ganglia cells, enabling the oviduct muscle to perform the needed oviposition movement (84). Ovary IIS may also modulate provisioning behaviors in social species where sterile workers feed offspring: in honey bees, workers with larger ovaries show a preference for pollen (used to make larval food) over nectar; genetic studies assessing variation in pollen preference have implicated the IIS pathway (85, 86).

## IIS regulation of adult polyphenisms

### Eusocial insect castes

IIS activity plays a critical developmental role across insects, affecting both juvenile and adult phenotypes (4). Here we highlight the developmental role of IIS in the context of adult polyphenisms, which are well-studied examples of nutrition-mediated behavioral variation in adult insects. For example, across independent evolutionary origins of eusociality, there is a common role for nutrition and IIS in caste determination, although the pathway is implemented differently among taxa (87–91). In honey bees, where colonies contain a single reproductive queen and thousands of sterile female worker bees, the queen larval diet increases IIS and leads to a spike in juvenile hormone (JH) production necessary for queen development (92–94). Later in development, queen IIS drops to worker-like levels (95), suggesting a transient increase in IIS/JH in queens gives rise to persistent effects at multiple levels of biological organization (93). While JH is produced in the corpora allata, it is unclear which tissues are involved in producing the upstream IIS signal and responding to IIS/JH (96).

IIS/JH signaling during larval stages could directly impact the development of the brain and/or other tissues that communicate with the brain throughout adulthood. In honey bees, IRS expression during development is responsible for differentiating queen and worker ovaries, but additional variation in IRS expression throughout adulthood also underpins behavior-relevant variation in ovary size among workers (85, 86, 97). For example, among workers, there is evidence that ovary size modulates the response to social pheromones (98). Enlarged ovaries are associated with increased octopamine signaling in the brain (98); octopamine activates the IPCs and thus could modulate olfactory sensitivity through IIS (8). A similar mechanism appears in the clonal raider ant *Ooceraea biroi*, where adults can switch between ovary activated (reproductive) and ovary suppressed (brood care) phases. Larval pheromones suppress reproduction and promote brood care by inhibiting ILP expression in adult IPCs (89).

While it is unknown whether or how IIS/JH signaling impacts brain development, differences in IIS expression continue into adulthood in honey bees; queens have decreased brain IIS compared to workers (87). Other social species also show caste differences in brain IIS, but patterns vary. Reproductives have higher brain IIS compared to workers in a wasp (*Polistes candensis* 99), termite (*Cavitermes tuberosus* 100), earwig (*Forficula Auricularia* 101), and many ant species (20, 89, 102–106). IIS could be linked to different, specific functional outcomes in these diverse social species, for example, species-specific trade-offs among egg production, queen behavior, and lifespan (87). Resolving these relationships requires more detailed work, including assessment of the specific mechanisms activated by IIS. For example, in reproductives of the ant *Harpegnathos saltator*, brain produced ILPs activate MAPK in the fat body and ovaries, but not the PI3K/Akt pathway (20), while ovarian activation of PI3K/Akt signaling occurs in other ant species (102, 103). These different responses to ILPs in the ovaries could mediate divergent phenotypic outcomes.

The unresolved complexities in IIS continue when looking among members of the worker caste in social insects. Honey bee workers show dietary and physiological changes corresponding to adult age-related behavioral shifts (“age polyethism”), including a massive loss of lipid stores in the fat body associated with the transition from nursing to foraging behaviors (107). As the fat body shrinks during aging, increased ILP production leads to increased JH and behavioral changes (97, 108–111). However, while older workers have higher whole-body IIS activity compared to younger workers, they have higher brain IIS (112) but lower fat body IIS (113). IIS activity differences could also correspond to tissue-specific divergence in downstream pathways. For example, a brain biomarker for honey bee foraging behavior is a extracellular signal-regulated kinase (ERK), a member of the MAPK pathway (114), which has been associated with learning and memory processes in the context of food acquisition (67). In contrast, in the fat body, IRS (the PI3K/Akt pathway) is activated in nurse bees who consume an amino acid rich diet compared to foragers; decreased IRS/IIS signaling results in precocious foraging (113). Thus, two different IIS downstream pathways in two different tissues both contribute to the same phenotypic outcome. Other honey bee species, the wasp *Polistes metricus*, and the ant *Temnothorax longispinosus* show similar age- and tissue-related patterns (88, 115–117), while the bumble bee *Bombus terrestris*, stingless bee *Tetragonisca angustula*, and ant *Solenopsis invicta* show the opposite, at least in terms of age patterns (118–120). The mechanistic implications of these complexities remain unclear.

Notably, many studies in eusocial insects use gene expression data exclusively to implicate IIS in caste differences. These data do not necessarily reflect circulating ILP levels or the quantity of stored ILPs that could be released to activate IIS. More work examining protein interactions and phosphorylation downstream of ILP receptor binding is necessary to validate and interpret the role of IIS in the context of behavioral differences between queens and workers or among workers.

### Wing length and weapon size polyphenisms

Juvenile nutrition and IIS activity are involved in the development of discrete adult polyphenisms in wing length in some hemipterans and weapon size in some coleopterans. When food quality is low, some hemipterans produce long-winged morphs that disperse at a cost to fecundity (121). As hemimetabolous insects, the switch between morphs can happen until the last nymphal instar, allowing for rapid response to environmental conditions (121). IIS patterns and wing morph expression are similar across several species: in soapberry bugs (*Jadera haematoloma*), linden bugs (*Pyrrhocoris apterus*), and pea aphids (*Acyrthosiphon pisum*), high quality food or low population densities lead to elevated IIS activity (inferred by pathway manipulation and gene expression data) and the development of wingless morphs (30, 51, 122, 123). However, in the brown planthopper (*Nilaparvata lugens*), this pattern is generally reversed (31, 124). Downstream mechanisms could include GSK3, which is associated with wing deformities (125, 126). Wing tissues are particularly sensitive to ILPs and variation in IIS does not affect allometry or growth in other tissues (121). This tissue specificity extends beyond species with conspicuous polyphenisms, e.g., *D. melanogaster* and the tobacco hornworm *Manduca sexta.* Thus, developmental nutrition may have other subtle effects on adult flight, dispersal phenotypes, and reproductive capacity across diverse species (19, 127).

In *Scarabaeidae* beetles, exaggerated male weapons like horns are common. Males with high-quality larval nutrition have large weapons and engage in male-male fighting over mates, while males with poor nutrition have small or no weapons and rely on sneaker tactics (10). As in the wing example, other tissues are unaffected by variation in ILPs. When nutrition is high-quality, ILPs drive weapon tissue proliferation through InR activation (128, 129). Without these signals, the transcription factor FOXO stops cell proliferation and the development of weapon structures (130). There is some interesting variation in how IIS acts in different beetle species. In the rhinoceros beetle *Trypoxylus dichotomus*, InR knockdown results in greatly diminished horns (130). In contrast, InR knockdown has no effect on horn growth in the dung beetle *Onthophagus nigriventris*, but FOXO knockdown suppresses growth in both the horns and genitalia (10, 29, 128, 131). IIS has also been implicated in more subtle variation in flight and fighting capabilities in bark beetles (*Dendroctonus ponderosae*) and crickets (*Gryllus assimilis* and *Gryllus firmus* 5, 132–134), suggesting it may play a more generalized role in competition-related behavior and polymorphisms.

In the dimorphic horned beetle examples, developmental IIS leads to differences in adult morphology and behavior, but it is unclear whether IIS exerts organizational effects on the brain during development, or continuously regulates adult behavioral differences. For example, variation in *doublesex* expression and serotonin levels in the adult brain predict differences in aggression across dimorphic males (135, 136). *Doublesex* is a target of IIS in developing horn tissues and serotonin impacts the body size threshold that distinguishes the horn morphs (29, 137), but it is unknown whether IIS regulates either mechanism in the adult brain. Similarly, in the pea aphid, differential ILP expression between nymphal winged and wingless individuals occurs in the thorax, but ILPs are also differentially expressed in the brain and thorax during adulthood, suggesting further phenotypic impacts (51, 138). Understanding the relationships in activity of IIS across the life stages could lead to new insights about the evolution and regulation of phenotypic plasticity. IIS appears to integrate environmental cues over the lifetime to modulate behavioral expression, and as such, it could serve as a mechanism that impacts the duration of environmental effects (139, 140).

## Discussion

IIS’s role in communicating nutritional state and regulating feeding behaviors has been elaborated over evolutionary time to coordinate reproductive physiology, courtship and mating behaviors, maternal provisioning behaviors, social insect caste differentiation, and the development and adult regulation of dimorphic dispersal and reproductive phenotypes.

Food choice and food-related locomotion are broadly associated with IIS, but there is substantial species-level variation in food cues, nutrients and preferences, locomotion patterns, and the ecological contexts that regulate foraging behaviors. Future studies could investigate the mechanistic bases of this species-level variation, in terms of how both internal state and external information modulate IIS and cause behavioral change. Insects present some particularly interesting and economically relevant contexts where IIS is essential to feeding behavior, including grasshopper (*Oedaleus asiaticus*) plague activity resulting from sub-optimal diets (141) or changes in feeding behavior due to crowding in armyworms (*Mythimna separata* 142). Examining IIS activation, including ILP production and modes of action in the brain across diverse taxa is critical to understanding the evolution of IIS and may also highlight new tools for pest control.

Substantial gaps remain in understanding the role of IIS in coordinating activities between the brain and peripheral tissues. These mechanisms are diverse and context dependent even in well studied species like *D. melanogaster* (15). However, certain emergent patterns may be conserved. For example, in *D. melanogaster*, different ILPs are responsible for within and cross-tissue signaling. ILP number varies greatly among taxa (143), possibly reflecting the diversity of contexts requiring IIS regulation, or the tissues involved. Most species have 1 or 2 InRs that activate tissue-specific downstream targets (144) but the mechanisms that allow specificity in downstream interactions, including how limited numbers of InR receptors give rise to diverse effects from numerous peptides, are still mostly unknown (55). While the most-studied model species *D. melanogaster* has only one InR, many other species have two, and Blattodea three, which can lead to novel relationships and interactions that should be studied further (144). For example, in the brown planthopper, InR2 directly inhibits InR1 during wing morph development, while the third Blattodea receptor is hypothesized to have a role in social termite evolution (124, 144).

Identifying the downstream pathways activated specifically by IIS is challenging as many of them can be affected by several other signaling pathways (20, 23, 145–147). This is especially problematic in non-model organisms where genetic tools and experimental approaches to manipulate ILP abundance are not well-developed. It is also important to consider the possibility that some peptides identified as insulin-like may belong to other peptide classes (e.g., IGF-like), which could suggest divergent downstream effects (20, 38, 105). Future studies could address these complexities by at least elaborating on the details of tissue-specific IIS and confirming the involvement of IIS using direct measures of ILP abundance and scaffolding or phosphorylation state of downstream targets (24).

Another compelling pattern that emerges from eusocial caste differentiation is that IIS is used to integrate cues associated with seasonal timing and other abiotic factors. For example, in the social paper wasp *Polistes metricus*, late season larvae become reproductive gynes that will overwinter and establish new nests the following year. As such, larvae are fed more and have activated IIS (148). The ant *Pogonomurmex rugosus* can only produce new queens after the original queen has hibernated, a transition caused by environmental signals like temperature that induce numerous physiological and behavioral changes in queens, including decreased metabolism and feeding. Hibernated queens have increased ILP expression, which increases the amount of vitellogenin deposited in eggs leading to new queen production (149). These provide additional examples of the ways in which IIS has been co-opted in novel contexts associated with nutrition variation.

Despite broad connections between IIS and behavior, mechanistic work outside of *D. melanogaster* remains limited. More diverse functional information could elucidate the conserved and divergent aspects of IIS among species and contexts, for example, in terms of where ILPs originate in the body (59), or how the different IIS components interact with each other (121). Our current model systems have highly derived phenotypes that may hinder attempts to form generalizable hypotheses. Broadening work in other taxa will also help explain why IIS is inconsistently used to regulate the same phenotypes across species (89). For example, some fig wasp species have winged and wingless males (150) that differ in aggression and weapon size (151). Although these phenotypes resemble the bugs and beetles discussed above, no link has been made to IIS or nutrition. Is this an independent evolutionary event with repeated co-option of the IIS pathway? Comparative investigations of the evolutionary origins of phenotypes like polyphenisms could help determine whether IIS is a “toolkit pathway” that has been repeatedly deployed over evolutionary time to give rise to similar phenotypes (120, 152). Its ubiquity among species and behaviors suggests this could be the case.

## Author contributions

AW: Conceptualization, Writing – original draft, Writing – review & editing. CR: Conceptualization, Funding acquisition, Supervision, Writing – original draft, Writing – review & editing.
